# 4-[4-(Hept­yloxy)benzo­yloxy]phenyl 2-oxo-7-tri­fluoro­methyl-2*H*-chromene-3-carboxyl­ate

**DOI:** 10.1107/S1600536813020679

**Published:** 2013-07-31

**Authors:** H. C. Devarajegowda, B. S. Palakshamurthy, H. N. Harishkumar, P. A. Suchetan, S. Sreenivasa

**Affiliations:** a Department of Physics, Yuvaraja’s College (Constituent College), University of Mysore Mysore, Karnataka 570 005, India; b Department of Chemistry Kuvempu University, Shankaraghatta Shimoga, Karnataka, India; c Department of Studies and Research in Chemistry, U.C.S, Tumkur University, Tumkur, Karnataka 572 103, India; d Department of Studies and Research in Chemistry, Tumkur University, Tumkur, Karnataka 572 103, India

## Abstract

The title compound, C_31_H_27_F_3_O_7_, is a liquid crystal and exhibits enanti­otropic SmA and nematic phase transitions. In the crystal, the the 2*H*-chromene ring system makes dihedral angles of 54.46 (17) and 7.79 (16)°, respectively, with the central benzene ring and 4-(hept­yloxy)benzene ring. The three F atoms of the –CF_3_ group are disordered over two sets of sites, with an occupancy ratio of 0.62 (3):0.38 (3). The crystal structre features two pairs of C—H⋯O hydrogen bonds, which form inversion dimers and generate *R*_2_^2^(10) and *R*_2_^2^(30) ring patterns. C—H⋯O inter­actions along [100] and C—H⋯π inter­actions futher consolidate the packing, leading to a three-dimensional network.

## Related literature

For similar structures, see: Palakshamurthy, Sreenivasa *et al.* (2013 ▶), Palakshamurthy, Devarajegowda *et al.* (2013 ▶). For graph-set notation for hydrogen bonds, see: Bernstein *et al.* (1995 ▶).
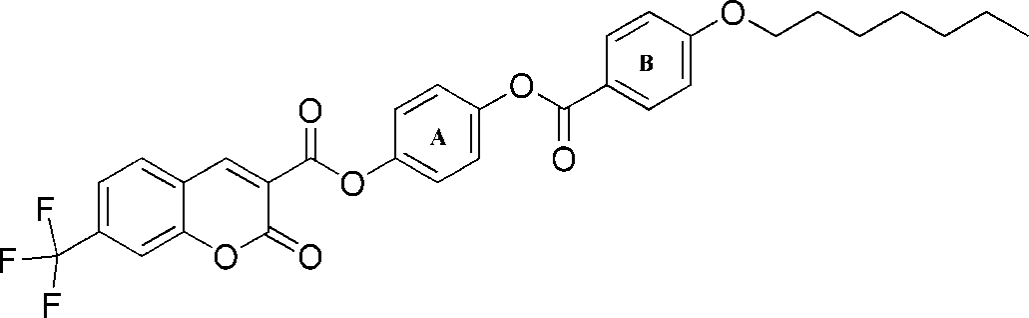


## Experimental

### 

#### Crystal data

C_31_H_27_F_3_O_7_
*M* *_r_* = 568.53
Triclinic, 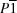

*a* = 5.6810 (3) Å
*b* = 16.036 (2) Å
*c* = 16.2954 (18) Å
α = 68.940 (12)°
β = 88.914 (6)°
γ = 88.486 (7)°
*V* = 1384.8 (3) Å^3^
*Z* = 2
Mo *K*α radiation
μ = 0.11 mm^−1^
*T* = 296 K
0.32 × 0.24 × 0.18 mm

#### Data collection

Bruker APEXII CCD area-detector diffractometer
Absorption correction: multi-scan (*SADABS*; Sheldrick, 2007 ▶) *T*_min_ = 0.966, *T*_max_ = 0.981
8822 measured reflections
4876 independent reflections
2837 reflections with *I* > 2σ(*I*)
*R*_int_ = 0.079

#### Refinement

*R*[*F* ^2^ > 2σ(*F* ^2^)] = 0.104
*wR*(*F* ^2^) = 0.316
*S* = 0.99
4876 reflections
399 parameters
H-atom parameters constrained
Δρ_max_ = 0.40 e Å^−3^
Δρ_min_ = −0.42 e Å^−3^

### 

Data collection: *APEX2* (Bruker, 2009 ▶); cell refinement: *APEX2* and *SAINT-Plus* (Bruker, 2009 ▶); data reduction: *SAINT-Plus* and *XPREP* (Bruker, 2009 ▶); program(s) used to solve structure: *SHELXS97* (Sheldrick, 2008 ▶); program(s) used to refine structure: *SHELXL97* (Sheldrick, 2008 ▶); molecular graphics: *Mercury* (Macrae *et al.*, 2008 ▶); software used to prepare material for publication: *SHELXL97*.

## Supplementary Material

Crystal structure: contains datablock(s) I, New_Global_Publ_Block. DOI: 10.1107/S1600536813020679/kj2229sup1.cif

Structure factors: contains datablock(s) I. DOI: 10.1107/S1600536813020679/kj2229Isup2.hkl

Supplementary material file. DOI: 10.1107/S1600536813020679/kj2229Isup3.cml

Additional supplementary materials:  crystallographic information; 3D view; checkCIF report

## Figures and Tables

**Table 1:** Hydrogen-bond geometry (Å, °) *Cg*1 and *Cg*2 are the centroids of the C12–C17 and C19–C24 rings, respectively.

*D*—H⋯*A*	*D*—H	H⋯*A*	*D*⋯*A*	*D*—H⋯*A*
C3—H3⋯O6^i^	0.93	2.53	3.313 (5)	142
C8—H8⋯O3^i^	0.93	2.44	3.277 (4)	150
C16—H16⋯O6^ii^	0.93	2.45	3.350 (5)	163
C14—H14⋯*Cg*2^iii^	0.93	2.81	3.517 (5)	133
C23—H23⋯*Cg*1^iv^	0.93	2.94	3.650 (5)	134

Symmetry codes: (i) 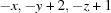; (ii) 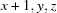; (iii) 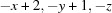; (iv) 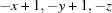.

**Fig. 1.**
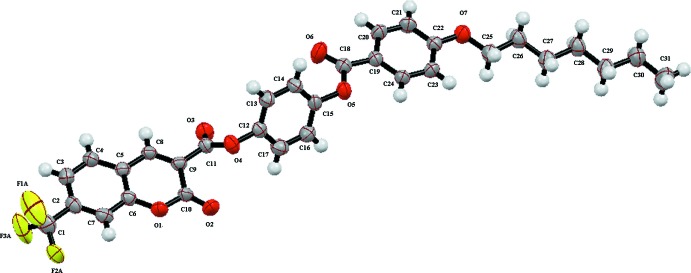
Molecular structure of the title compound, showing displacement ellipsoids drawn at the 50% probability level. Only the major component of the disordered CF3 group is shown.

**Fig. 2.**
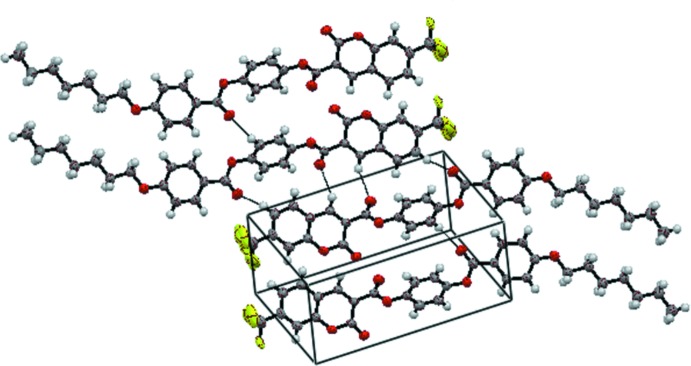
Crystal packing of the title compound with hydrogen bonds drawn as dashed lines.

**Fig. 3.**
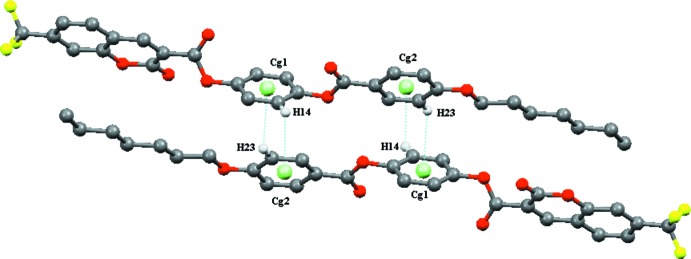
Packing of the title compound. C—H···π interactions are shown as dashed lines.

## supplementary crystallographic information

### Comment

As a part of our continued efforts to study the structure of coumarin based liquid crystals (LC), we report herein the crystal structure of 4-(4-(heptyloxy)benzoyloxy)phenyl 7-(trifluoromethyl)-2-oxo-2*H*-chromene-3-carboxylate (I), and its comparision with 4-(decyloxy)phenyl 7-(trifluoromethyl)- 2-oxo-2*H*-chromene-3-carboxylate (II), 4-(octyloxy)phenyl 2-oxo-2*H*-chromene-3 –carboxylate (III) (Palakshamurthy, Sreenivasa *et al.*, 2013; Palakshamurthy, Devarajegowda *et al.*, 2013). The title compound, C_31_H_27_F_3_O_7_, is a liquid crystal (LC) exhibiting enantiotropic SmA, nematic phase transitions at 520.2(*2.0*), 522.7(*2.7*) on heating and at 519.6(*2.0*), 522.1(*2.9*) on cooling [The transition temperature in K and the associated enthalpy values in kJ mol-1 (in italics)] The asymmetric unit of 4-(4-(heptyloxy)benzoyloxy)phenyl 7-(trifluoromethyl)-2-oxo-2*H*-chromene-3-carboxylate is shown in Fig.1.The three F atoms of the –CF3 group are disordered over two sets of sites with occupancy factors 0.62 (3):0.38 (3).The dihedral angle between the 2*H*-chromene ring and the benzene ring A in the compound I is 54.46 (17)°, compared to the observed values of 62.97 (2)°, 21.11 (1)° in compounds II and III respectively. The crystal structre is stabilized by two pairs of C8—H8···O3 and C3—H3···O6 hydrogen bonds form inversion dimers and generate *R*_2_^2^(10) and *R*_2_^2^(30) ring patterns respectivly (Bernstein *et al.*, 1995). The C16—H16···O6 contact and C—H···*Cg*1 (centroid of C12—C17) and C—H···*Cg*2 (centroid of C19—C24) interactions further strengthen the packing (Fig. 2, Fig.3).

### Experimental

A mixture of 7-(trifluoromethyl)-2-oxo-2H-chromene-3-carboxylic acid (258 mg, 0.01 mmol), 4-hydroxyphenyl 4-(heptyloxy)benzoate (358 mg, 0.01 mmol), N,N-dicyclohexylcarbodiimide (DCC) (210 mg, 0.012 mmol) and catalytic quantity of dimethylaminopyridimidine with anhydrous tetrahydrofuran (5 ml) was stirred for 24hrs at room temperature. The *N,N*-dicyclohexylurea formed was filtered off and the filtrate was diluted with dichloromethane (25 ml). This solution was washed successively with water (2 x 30 ml), 5% aqueous acetic acid (3 x 50 ml), water (3 x 50ml) and was then dried (Na_2_SO_4_). The residue obtained on removal of solvent was chromatographed on silica gel and eluted with chloroform as an eluent. Removal of solvent from the eluate afforded a white solid material which was crystallized repeatedly from ethanol to get colourless blocks.

### Refinement

The H atoms bound to carbon were positioned with idealized geometry using a riding model with d(C–H) = 0.93- 0.97 Å. All C–H atoms were refined with isotropic displacement parameters set to 1.2–1.5 *U*_eq_(C). The F1, F2, and F3 fluorine atoms of the –CF3 group were disordered over two sites and refined with site occupancy factors 0.62 (3):0.38 (3).

### Crystal data

**Table:**

C_31_H_27_F_3_O_7_	*F*(000) = 592
*M**_r_* = 568.53	Blocks
Triclinic, *P*1	*D*_x_ = 1.363 Mg m^−^^3^
Hall symbol: -P 1	Melting point: 434 K
*a* = 5.6810 (3) Å	Mo *K*α radiation, λ = 0.71073 Å
*b* = 16.036 (2) Å	Cell parameters from 2837 reflections
*c* = 16.2954 (18) Å	θ = 2.5–25°
α = 68.940 (12)°	µ = 0.11 mm^−^^1^
β = 88.914 (6)°	*T* = 296 K
γ = 88.486 (7)°	Block, colourless
*V* = 1384.8 (3) Å^3^	0.32 × 0.24 × 0.18 mm
*Z* = 2	

### Data collection

**Table:**

Bruker APEXII CCD area-detector diffractometer	4876 independent reflections
Radiation source: fine-focus sealed tube	2837 reflections with *I* > 2σ(*I*)
Graphite monochromator	*R*_int_ = 0.079
Detector resolution: 1.03 pixels mm^-1^	θ_max_ = 25.0°, θ_min_ = 2.5°
phi and ω scans	*h* = −6→6
Absorption correction: multi-scan (*SADABS*; Sheldrick, 2007)	*k* = −19→18
*T*_min_ = 0.966, *T*_max_ = 0.981	*l* = −19→17
8822 measured reflections	

### Refinement

**Table:**

Refinement on *F*^2^	Primary atom site location: structure-invariant direct methods
Least-squares matrix: full	Secondary atom site location: difference Fourier map
*R*[*F*^2^ > 2σ(*F*^2^)] = 0.104	Hydrogen site location: inferred from neighbouring sites
*wR*(*F*^2^) = 0.316	H-atom parameters constrained
*S* = 0.99	*w* = 1/[σ^2^(*F*_o_^2^) + (0.1918*P*)^2^] where *P* = (*F*_o_^2^ + 2*F*_c_^2^)/3
4876 reflections	(Δ/σ)_max_ < 0.001
399 parameters	Δρ_max_ = 0.40 e Å^−^^3^
0 restraints	Δρ_min_ = −0.42 e Å^−^^3^
0 constraints	

### Special details

**Table:**

Geometry. All e.s.d.'s (except the e.s.d. in the dihedral angle between two l.s. planes) are estimated using the full covariance matrix. The cell e.s.d.'s are taken into account individually in the estimation of e.s.d.'s in distances, angles and torsion angles; correlations between e.s.d.'s in cell parameters are only used when they are defined by crystal symmetry. An approximate (isotropic) treatment of cell e.s.d.'s is used for estimating e.s.d.'s involving l.s. planes.
Refinement. Refinement of *F*^2^ against ALL reflections. The weighted *R*-factor *wR* and goodness of fit *S* are based on *F*^2^, conventional *R*-factors *R* are based on *F*, with *F* set to zero for negative *F*^2^. The threshold expression of *F*^2^ > σ(*F*^2^) is used only for calculating *R*-factors(gt) *etc*. and is not relevant to the choice of reflections for refinement. *R*-factors based on *F*^2^ are statistically about twice as large as those based on *F*, and *R*- factors based on ALL data will be even larger.

### Fractional atomic coordinates and isotropic or equivalent isotropic displacement parameters (Å^2^)

**Table:**

	*x*	*y*	*z*	*U*_iso_*/*U*_eq_	Occ. (<1)
C1	0.5018 (9)	1.1195 (3)	0.0428 (3)	0.0728 (13)	
C2	0.4321 (7)	1.0781 (3)	0.1360 (3)	0.0538 (10)	
C3	0.2160 (7)	1.1040 (3)	0.1631 (3)	0.0580 (10)	
H3	0.1172	1.1448	0.1225	0.070*	
C4	0.1518 (6)	1.0687 (2)	0.2499 (3)	0.0519 (10)	
H4	0.0076	1.0854	0.2679	0.062*	
C5	0.2991 (6)	1.0080 (2)	0.3119 (3)	0.0459 (9)	
C6	0.5150 (6)	0.9843 (2)	0.2825 (3)	0.0470 (9)	
C7	0.5806 (7)	1.0184 (3)	0.1946 (3)	0.0566 (10)	
H7	0.7228	1.0009	0.1758	0.068*	
C8	0.2469 (6)	0.9689 (2)	0.4027 (2)	0.0450 (9)	
H8	0.1043	0.9838	0.4234	0.054*	
C9	0.3928 (5)	0.9114 (2)	0.4601 (2)	0.0442 (8)	
C10	0.6252 (6)	0.8895 (2)	0.4300 (3)	0.0485 (9)	
C11	0.3301 (6)	0.8718 (2)	0.5537 (2)	0.0446 (8)	
C12	0.3494 (6)	0.7435 (2)	0.6811 (3)	0.0465 (9)	
C13	0.1534 (7)	0.6919 (3)	0.7119 (3)	0.0547 (10)	
H13	0.0419	0.6868	0.6730	0.066*	
C14	0.1256 (6)	0.6483 (3)	0.8005 (3)	0.0551 (10)	
H14	−0.0056	0.6133	0.8222	0.066*	
C15	0.2911 (6)	0.6561 (2)	0.8575 (3)	0.0506 (9)	
C16	0.4882 (7)	0.7071 (3)	0.8258 (3)	0.0566 (10)	
H16	0.6005	0.7120	0.8645	0.068*	
C17	0.5167 (6)	0.7501 (3)	0.7371 (3)	0.0591 (11)	
H17	0.6499	0.7838	0.7151	0.071*	
C18	0.0998 (6)	0.6353 (3)	0.9943 (3)	0.0527 (10)	
C19	0.1256 (6)	0.5916 (2)	1.0887 (3)	0.0481 (9)	
C20	−0.0520 (6)	0.6051 (3)	1.1441 (3)	0.0552 (10)	
H20	−0.1829	0.6408	1.1195	0.066*	
C21	−0.0360 (6)	0.5673 (3)	1.2323 (3)	0.0579 (10)	
H21	−0.1572	0.5764	1.2676	0.069*	
C22	0.1589 (6)	0.5149 (2)	1.2712 (3)	0.0482 (9)	
C23	0.3373 (6)	0.5008 (2)	1.2179 (3)	0.0521 (9)	
H23	0.4680	0.4652	1.2430	0.062*	
C24	0.3204 (6)	0.5393 (2)	1.1281 (2)	0.0491 (9)	
H24	0.4419	0.5303	1.0930	0.059*	
C25	0.3583 (7)	0.4275 (3)	1.4026 (3)	0.0586 (10)	
H25A	0.4969	0.4643	1.3873	0.070*	
H25B	0.3840	0.3777	1.3827	0.070*	
C26	0.3225 (7)	0.3932 (3)	1.4994 (3)	0.0633 (11)	
H26A	0.1929	0.3520	1.5147	0.076*	
H26B	0.2803	0.4427	1.5181	0.076*	
C27	0.5406 (8)	0.3461 (3)	1.5475 (3)	0.0638 (11)	
H27A	0.5867	0.2993	1.5254	0.077*	
H27B	0.6671	0.3886	1.5333	0.077*	
C28	0.5177 (7)	0.3050 (3)	1.6459 (3)	0.0624 (11)	
H28A	0.3880	0.2639	1.6609	0.075*	
H28B	0.4810	0.3517	1.6691	0.075*	
C29	0.7420 (8)	0.2550 (3)	1.6890 (3)	0.0666 (12)	
H29A	0.7791	0.2093	1.6645	0.080*	
H29B	0.8705	0.2966	1.6736	0.080*	
C30	0.7303 (8)	0.2118 (3)	1.7865 (3)	0.0759 (14)	
H30A	0.5981	0.1719	1.8024	0.091*	
H30B	0.7017	0.2576	1.8116	0.091*	
C31	0.9507 (10)	0.1598 (5)	1.8258 (4)	0.108 (2)	
H31A	0.9800	0.1141	1.8014	0.162*	
H31B	0.9312	0.1327	1.8884	0.162*	
H31C	1.0814	0.1994	1.8125	0.162*	
O1	0.6675 (4)	0.92522 (17)	0.34057 (18)	0.0549 (7)	
O2	0.7777 (5)	0.8451 (2)	0.4762 (2)	0.0744 (10)	
O3	0.2319 (5)	0.91399 (18)	0.5935 (2)	0.0646 (8)	
O6	−0.0516 (5)	0.6888 (2)	0.9571 (2)	0.0797 (10)	
F1	0.656 (4)	1.0696 (9)	0.0209 (8)	0.130 (5)	0.62 (3)
F2	0.600 (4)	1.1943 (10)	0.0239 (8)	0.120 (5)	0.62 (3)
F3	0.346 (2)	1.1281 (18)	−0.0147 (10)	0.139 (7)	0.62 (3)
F1A	0.364 (7)	1.091 (2)	−0.0042 (18)	0.149 (11)	0.38 (3)
F2A	0.731 (3)	1.100 (2)	0.0245 (10)	0.143 (11)	0.38 (3)
F3A	0.471 (6)	1.2076 (13)	0.0083 (16)	0.140 (10)	0.38 (3)
O4	0.3862 (5)	0.78518 (16)	0.59002 (17)	0.0557 (7)	
O7	0.1577 (5)	0.47897 (19)	1.35995 (19)	0.0611 (8)	
O5	0.2751 (4)	0.61051 (18)	0.94774 (17)	0.0576 (7)	

### Atomic displacement parameters (Å^2^)

**Table:**

	*U*^11^	*U*^22^	*U*^33^	*U*^12^	*U*^13^	*U*^23^
C1	0.088 (3)	0.074 (3)	0.056 (3)	−0.002 (3)	0.008 (3)	−0.023 (2)
C2	0.063 (2)	0.059 (2)	0.038 (2)	−0.0074 (18)	0.0065 (17)	−0.0160 (18)
C3	0.057 (2)	0.058 (2)	0.051 (3)	−0.0010 (18)	−0.0035 (18)	−0.0096 (19)
C4	0.0464 (19)	0.052 (2)	0.051 (2)	0.0005 (16)	0.0053 (16)	−0.0110 (17)
C5	0.0412 (17)	0.0395 (18)	0.054 (2)	−0.0005 (14)	0.0032 (15)	−0.0130 (16)
C6	0.0469 (19)	0.0453 (18)	0.047 (2)	0.0002 (15)	0.0064 (16)	−0.0154 (17)
C7	0.052 (2)	0.061 (2)	0.056 (3)	−0.0052 (18)	0.0122 (18)	−0.021 (2)
C8	0.0376 (17)	0.0443 (18)	0.050 (2)	0.0004 (14)	0.0087 (15)	−0.0137 (17)
C9	0.0366 (17)	0.0465 (19)	0.049 (2)	0.0017 (14)	0.0058 (15)	−0.0177 (16)
C10	0.0400 (18)	0.0465 (19)	0.051 (2)	0.0026 (15)	0.0101 (16)	−0.0080 (16)
C11	0.0453 (18)	0.0428 (18)	0.045 (2)	0.0025 (14)	0.0046 (15)	−0.0157 (16)
C12	0.052 (2)	0.0383 (17)	0.047 (2)	0.0065 (15)	0.0048 (16)	−0.0134 (16)
C13	0.056 (2)	0.057 (2)	0.048 (2)	−0.0072 (17)	−0.0036 (17)	−0.0152 (18)
C14	0.051 (2)	0.061 (2)	0.047 (2)	−0.0108 (17)	0.0033 (17)	−0.0123 (18)
C15	0.052 (2)	0.049 (2)	0.041 (2)	0.0089 (16)	0.0031 (16)	−0.0065 (16)
C16	0.050 (2)	0.063 (2)	0.055 (3)	0.0000 (17)	−0.0044 (17)	−0.0198 (19)
C17	0.053 (2)	0.052 (2)	0.069 (3)	−0.0066 (17)	0.0071 (19)	−0.018 (2)
C18	0.050 (2)	0.056 (2)	0.048 (2)	0.0028 (17)	0.0021 (17)	−0.0138 (18)
C19	0.0479 (19)	0.0474 (19)	0.043 (2)	0.0010 (15)	0.0024 (16)	−0.0100 (16)
C20	0.0459 (19)	0.062 (2)	0.053 (2)	0.0130 (17)	−0.0005 (17)	−0.0158 (19)
C21	0.050 (2)	0.071 (2)	0.050 (2)	0.0088 (18)	0.0098 (17)	−0.020 (2)
C22	0.0475 (19)	0.0475 (19)	0.046 (2)	−0.0013 (15)	0.0033 (16)	−0.0129 (16)
C23	0.0414 (18)	0.055 (2)	0.055 (3)	0.0092 (15)	−0.0008 (16)	−0.0157 (18)
C24	0.0448 (18)	0.058 (2)	0.041 (2)	0.0042 (15)	0.0044 (15)	−0.0146 (17)
C25	0.062 (2)	0.064 (2)	0.049 (3)	0.0108 (18)	0.0024 (18)	−0.0188 (19)
C26	0.073 (3)	0.069 (3)	0.045 (3)	0.003 (2)	0.004 (2)	−0.017 (2)
C27	0.072 (3)	0.062 (2)	0.056 (3)	0.007 (2)	0.000 (2)	−0.021 (2)
C28	0.074 (3)	0.066 (2)	0.044 (2)	0.008 (2)	0.002 (2)	−0.0174 (19)
C29	0.077 (3)	0.068 (3)	0.052 (3)	0.010 (2)	0.000 (2)	−0.021 (2)
C30	0.076 (3)	0.084 (3)	0.056 (3)	0.006 (2)	−0.003 (2)	−0.013 (2)
C31	0.088 (3)	0.150 (5)	0.066 (4)	0.036 (3)	−0.003 (3)	−0.017 (4)
O1	0.0460 (13)	0.0605 (16)	0.0526 (17)	0.0086 (11)	0.0122 (12)	−0.0149 (13)
O2	0.0458 (15)	0.082 (2)	0.072 (2)	0.0167 (14)	0.0041 (14)	−0.0008 (17)
O3	0.0740 (18)	0.0595 (16)	0.0583 (19)	0.0182 (13)	0.0114 (14)	−0.0206 (14)
O6	0.0733 (19)	0.097 (2)	0.059 (2)	0.0362 (17)	−0.0088 (15)	−0.0179 (17)
F1	0.161 (12)	0.120 (7)	0.103 (7)	0.017 (7)	0.066 (7)	−0.040 (5)
F2	0.191 (13)	0.085 (7)	0.077 (5)	−0.064 (8)	0.047 (7)	−0.018 (5)
F3	0.124 (7)	0.211 (18)	0.049 (4)	−0.048 (8)	−0.016 (4)	−0.002 (8)
F1A	0.26 (3)	0.142 (16)	0.059 (12)	−0.055 (13)	0.000 (11)	−0.048 (12)
F2A	0.077 (7)	0.23 (3)	0.047 (6)	0.004 (10)	0.025 (5)	0.041 (10)
F3A	0.194 (19)	0.086 (8)	0.089 (10)	0.059 (13)	0.059 (12)	0.026 (7)
O4	0.0720 (17)	0.0430 (14)	0.0482 (17)	0.0063 (12)	0.0114 (13)	−0.0126 (12)
O7	0.0623 (16)	0.0725 (18)	0.0451 (17)	0.0126 (13)	0.0021 (12)	−0.0179 (14)
O5	0.0637 (16)	0.0617 (16)	0.0388 (15)	0.0136 (12)	0.0025 (12)	−0.0089 (12)

### Geometric parameters (Å, º)

**Table:**

C1—F2	1.268 (9)	C17—H17	0.9300
C1—F3	1.268 (14)	C18—O6	1.204 (4)
C1—F1	1.302 (12)	C18—O5	1.376 (5)
C1—F1A	1.31 (3)	C18—O5	1.376 (5)
C1—F3A	1.328 (17)	C18—C19	1.451 (5)
C1—F2A	1.379 (18)	C19—C24	1.390 (5)
C1—C2	1.473 (6)	C19—C20	1.405 (6)
C2—C7	1.368 (5)	C20—C21	1.349 (5)
C2—C3	1.399 (6)	C20—H20	0.9300
C3—C4	1.367 (6)	C21—C22	1.389 (5)
C3—H3	0.9300	C21—H21	0.9300
C4—C5	1.397 (5)	C22—O7	1.351 (5)
C4—H4	0.9300	C22—C23	1.389 (6)
C5—C6	1.401 (5)	C23—C24	1.373 (5)
C5—C8	1.412 (5)	C23—H23	0.9300
C6—O1	1.373 (4)	C24—H24	0.9300
C6—C7	1.385 (5)	C25—O7	1.425 (4)
C7—H7	0.9300	C25—C26	1.484 (5)
C8—C9	1.334 (5)	C25—H25A	0.9700
C8—H8	0.9300	C25—H25B	0.9700
C9—C11	1.467 (5)	C26—C27	1.512 (6)
C9—C10	1.477 (5)	C26—H26A	0.9700
C10—O2	1.197 (4)	C26—H26B	0.9700
C10—O1	1.379 (5)	C27—C28	1.503 (6)
C11—O3	1.212 (5)	C27—H27A	0.9700
C11—O4	1.332 (4)	C27—H27B	0.9700
C12—C17	1.362 (5)	C28—C29	1.528 (6)
C12—C13	1.381 (5)	C28—H28A	0.9700
C12—O4	1.405 (4)	C28—H28B	0.9700
C13—C14	1.368 (5)	C29—C30	1.488 (6)
C13—H13	0.9300	C29—H29A	0.9700
C14—C15	1.372 (5)	C29—H29B	0.9700
C14—H14	0.9300	C30—C31	1.505 (7)
C15—C16	1.383 (5)	C30—H30A	0.9700
C15—O5	1.390 (4)	C30—H30B	0.9700
C15—O5	1.390 (4)	C31—H31A	0.9600
C16—C17	1.368 (6)	C31—H31B	0.9600
C16—H16	0.9300	C31—H31C	0.9600
			
F2—C1—F3	107.6 (10)	O6—C18—O5	121.0 (4)
F2—C1—F1	104.7 (8)	O6—C18—C19	126.5 (4)
F3—C1—F1	100.0 (12)	O5—C18—C19	112.5 (3)
F2—C1—F1A	130.1 (13)	O5—C18—C19	112.5 (3)
F1—C1—F1A	82.3 (17)	C24—C19—C20	117.6 (4)
F3—C1—F3A	77.9 (13)	C24—C19—C18	123.9 (4)
F1—C1—F3A	128.4 (10)	C20—C19—C18	118.5 (3)
F1A—C1—F3A	103.1 (17)	C21—C20—C19	121.2 (3)
F2—C1—F2A	77.5 (10)	C21—C20—H20	119.4
F3—C1—F2A	118.2 (12)	C19—C20—H20	119.4
F1A—C1—F2A	107.4 (19)	C20—C21—C22	120.9 (4)
F3A—C1—F2A	107.3 (12)	C20—C21—H21	119.6
F2—C1—C2	114.1 (6)	C22—C21—H21	119.6
F3—C1—C2	117.8 (8)	O7—C22—C23	124.3 (3)
F1—C1—C2	111.0 (6)	O7—C22—C21	116.5 (3)
F1A—C1—C2	108.6 (14)	C23—C22—C21	119.1 (4)
F3A—C1—C2	115.2 (10)	C24—C23—C22	119.9 (3)
F2A—C1—C2	114.4 (7)	C24—C23—H23	120.1
C7—C2—C3	121.3 (4)	C22—C23—H23	120.1
C7—C2—C1	120.2 (4)	C23—C24—C19	121.4 (4)
C3—C2—C1	118.4 (4)	C23—C24—H24	119.3
C4—C3—C2	119.3 (3)	C19—C24—H24	119.3
C4—C3—H3	120.3	O7—C25—C26	110.2 (3)
C2—C3—H3	120.3	O7—C25—H25A	109.6
C3—C4—C5	121.1 (4)	C26—C25—H25A	109.6
C3—C4—H4	119.4	O7—C25—H25B	109.6
C5—C4—H4	119.4	C26—C25—H25B	109.6
C4—C5—C6	118.0 (4)	H25A—C25—H25B	108.1
C4—C5—C8	124.7 (3)	C25—C26—C27	111.9 (4)
C6—C5—C8	117.3 (3)	C25—C26—H26A	109.2
O1—C6—C7	117.9 (3)	C27—C26—H26A	109.2
O1—C6—C5	120.6 (3)	C25—C26—H26B	109.2
C7—C6—C5	121.5 (3)	C27—C26—H26B	109.2
C2—C7—C6	118.7 (4)	H26A—C26—H26B	107.9
C2—C7—H7	120.7	C28—C27—C26	115.8 (4)
C6—C7—H7	120.7	C28—C27—H27A	108.3
C9—C8—C5	123.0 (3)	C26—C27—H27A	108.3
C9—C8—H8	118.5	C28—C27—H27B	108.3
C5—C8—H8	118.5	C26—C27—H27B	108.3
C8—C9—C11	121.1 (3)	H27A—C27—H27B	107.4
C8—C9—C10	119.8 (3)	C27—C28—C29	112.5 (4)
C11—C9—C10	119.1 (3)	C27—C28—H28A	109.1
O2—C10—O1	118.2 (3)	C29—C28—H28A	109.1
O2—C10—C9	125.7 (4)	C27—C28—H28B	109.1
O1—C10—C9	116.0 (3)	C29—C28—H28B	109.1
O3—C11—O4	123.4 (4)	H28A—C28—H28B	107.8
O3—C11—C9	122.8 (3)	C30—C29—C28	115.2 (4)
O4—C11—C9	113.7 (3)	C30—C29—H29A	108.5
C17—C12—C13	121.2 (4)	C28—C29—H29A	108.5
C17—C12—O4	119.0 (3)	C30—C29—H29B	108.5
C13—C12—O4	119.7 (3)	C28—C29—H29B	108.5
C14—C13—C12	119.1 (3)	H29A—C29—H29B	107.5
C14—C13—H13	120.5	C29—C30—C31	113.4 (5)
C12—C13—H13	120.5	C29—C30—H30A	108.9
C13—C14—C15	120.1 (3)	C31—C30—H30A	108.9
C13—C14—H14	120.0	C29—C30—H30B	108.9
C15—C14—H14	120.0	C31—C30—H30B	108.9
C14—C15—C16	120.4 (4)	H30A—C30—H30B	107.7
C14—C15—O5	122.2 (3)	C30—C31—H31A	109.5
C16—C15—O5	117.3 (3)	C30—C31—H31B	109.5
C14—C15—O5	122.2 (3)	H31A—C31—H31B	109.5
C16—C15—O5	117.3 (3)	C30—C31—H31C	109.5
C17—C16—C15	119.5 (4)	H31A—C31—H31C	109.5
C17—C16—H16	120.3	H31B—C31—H31C	109.5
C15—C16—H16	120.3	C6—O1—C10	123.1 (3)
C12—C17—C16	119.8 (3)	C11—O4—C12	117.5 (3)
C12—C17—H17	120.1	C22—O7—C25	118.3 (3)
C16—C17—H17	120.1	C18—O5—C15	118.6 (3)
O6—C18—O5	121.0 (4)		
			
F2—C1—C2—C7	−93.5 (14)	O5—C15—C16—C17	177.2 (3)
F3—C1—C2—C7	139.0 (15)	C13—C12—C17—C16	−1.9 (6)
F1—C1—C2—C7	24.5 (13)	O4—C12—C17—C16	−177.4 (3)
F1A—C1—C2—C7	113 (2)	C15—C16—C17—C12	1.0 (6)
F3A—C1—C2—C7	−132 (2)	O6—C18—C19—C24	−171.3 (4)
F2A—C1—C2—C7	−6.8 (19)	O5—C18—C19—C24	7.5 (5)
F2—C1—C2—C3	84.1 (14)	O5—C18—C19—C24	7.5 (5)
F3—C1—C2—C3	−43.4 (16)	O6—C18—C19—C20	6.4 (6)
F1—C1—C2—C3	−157.9 (12)	O5—C18—C19—C20	−174.8 (3)
F1A—C1—C2—C3	−69 (2)	O5—C18—C19—C20	−174.8 (3)
F3A—C1—C2—C3	46 (2)	C24—C19—C20—C21	−1.3 (6)
F2A—C1—C2—C3	170.8 (19)	C18—C19—C20—C21	−179.1 (3)
C7—C2—C3—C4	−0.2 (6)	C19—C20—C21—C22	1.1 (6)
C1—C2—C3—C4	−177.7 (4)	C20—C21—C22—O7	−179.2 (3)
C2—C3—C4—C5	0.6 (6)	C20—C21—C22—C23	−0.9 (6)
C3—C4—C5—C6	0.0 (5)	O7—C22—C23—C24	179.1 (3)
C3—C4—C5—C8	179.7 (3)	C21—C22—C23—C24	0.8 (6)
C4—C5—C6—O1	179.7 (3)	C22—C23—C24—C19	−1.1 (6)
C8—C5—C6—O1	0.0 (5)	C20—C19—C24—C23	1.3 (5)
C4—C5—C6—C7	−1.1 (5)	C18—C19—C24—C23	178.9 (3)
C8—C5—C6—C7	179.2 (3)	O7—C25—C26—C27	−174.3 (3)
C3—C2—C7—C6	−0.9 (6)	C25—C26—C27—C28	−177.1 (3)
C1—C2—C7—C6	176.6 (4)	C26—C27—C28—C29	177.3 (4)
O1—C6—C7—C2	−179.3 (3)	C27—C28—C29—C30	−179.4 (4)
C5—C6—C7—C2	1.6 (6)	C28—C29—C30—C31	177.3 (5)
C4—C5—C8—C9	−179.7 (3)	C7—C6—O1—C10	177.5 (3)
C6—C5—C8—C9	0.0 (5)	C5—C6—O1—C10	−3.3 (5)
C5—C8—C9—C11	−179.5 (3)	O2—C10—O1—C6	−173.3 (3)
C5—C8—C9—C10	2.8 (5)	C9—C10—O1—C6	5.9 (5)
C8—C9—C10—O2	173.4 (4)	O3—C11—O4—C12	−6.4 (5)
C11—C9—C10—O2	−4.3 (6)	C9—C11—O4—C12	175.1 (3)
C8—C9—C10—O1	−5.6 (5)	C17—C12—O4—C11	−83.4 (4)
C11—C9—C10—O1	176.7 (3)	C13—C12—O4—C11	101.0 (4)
C8—C9—C11—O3	−40.1 (5)	C23—C22—O7—C25	4.0 (5)
C10—C9—C11—O3	137.6 (4)	C21—C22—O7—C25	−177.7 (3)
C8—C9—C11—O4	138.4 (3)	C26—C25—O7—C22	−179.4 (3)
C10—C9—C11—O4	−43.9 (4)	O6—C18—O5—O5	0.0 (10)
C17—C12—C13—C14	1.4 (6)	C19—C18—O5—O5	0.0 (9)
O4—C12—C13—C14	176.9 (3)	O6—C18—O5—C15	7.6 (6)
C12—C13—C14—C15	0.0 (6)	O5—C18—O5—C15	0 (100)
C13—C14—C15—C16	−0.8 (6)	C19—C18—O5—C15	−171.3 (3)
C13—C14—C15—O5	−177.5 (4)	C16—C15—O5—O5	0.0 (8)
C13—C14—C15—O5	−177.5 (4)	C14—C15—O5—C18	−69.4 (5)
C14—C15—C16—C17	0.4 (6)	C16—C15—O5—C18	113.8 (4)
O5—C15—C16—C17	177.2 (3)	O5—C15—O5—C18	0 (100)

### Hydrogen-bond geometry (Å, º)

Cg1 and Cg2 are the centroids of the C12–C17 and C19–C24 rings, respectively.

**Table:**

*D*—H···*A*	*D*—H	H···*A*	*D*···*A*	*D*—H···*A*
C3—H3···O6^i^	0.93	2.53	3.313 (5)	142
C8—H8···O3^i^	0.93	2.44	3.277 (4)	150
C16—H16···O6^ii^	0.93	2.45	3.350 (5)	163
C24—H24···O5	0.93	2.45	2.759 (5)	100
C14—H14···*Cg*2^iii^	0.93	2.81	3.517 (5)	133
C23—H23···*Cg*1^iv^	0.93	2.94	3.650 (5)	134

Symmetry codes: (i) −*x*, −*y*+2, −*z*+1; (ii) *x*+1, *y*, *z*; (iii) −*x*+2, −*y*+1, −*z*; (iv) −*x*+1, −*y*+1, −*z*.
